# Automated classification of protein subcellular localization in immunohistochemistry images to reveal biomarkers in colon cancer

**DOI:** 10.1186/s12859-020-03731-y

**Published:** 2020-09-09

**Authors:** Zhen-Zhen Xue, Yanxia Wu, Qing-Zu Gao, Liang Zhao, Ying-Ying Xu

**Affiliations:** 1 School of Biomedical Engineering and Guangdong Provincial Key Laboratory of Medical Image Processing, Southern Medical University, Guangzhou, 510515 China; 2 Department of Pathology, Nanfang Hospital, Southern Medical University, Guangzhou, 510515 China; 3 Department of Clinical Pathology, Affiliated Hospital of Guangdong Medical University, Zhanjiang, 524000 China; 4 The First Affiliated Hospital of Xinxiang Medical University, Xinxiang, China; 5 Department of Pathology, School of Basic Medical Sciences, Southern Medical University, Guangzhou, 510515 China

**Keywords:** Bioimage processing, Bioinformatics, Machine learning, Protein subcellular location, Cancer biomarkers

## Abstract

**Background:**

Protein biomarkers play important roles in cancer diagnosis. Many efforts have been made on measuring abnormal expression intensity in biological samples to identity cancer types and stages. However, the change of subcellular location of proteins, which is also critical for understanding and detecting diseases, has been rarely studied.

**Results:**

In this work, we developed a machine learning model to classify protein subcellular locations based on immunohistochemistry images of human colon tissues, and validated the ability of the model to detect subcellular location changes of biomarker proteins related to colon cancer. The model uses representative image patches as inputs, and integrates feature engineering and deep learning methods. It achieves 92.69% accuracy in classification of new proteins. Two validation datasets of colon cancer biomarkers derived from published literatures and the human protein atlas database respectively are employed. It turns out that 81.82 and 65.66% of the biomarker proteins can be identified to change locations.

**Conclusions:**

Our results demonstrate that using image patches and combining predefined and deep features can improve the performance of protein subcellular localization, and our model can effectively detect biomarkers based on protein subcellular translocations. This study is anticipated to be useful in annotating unknown subcellular localization for proteins and discovering new potential location biomarkers.

## Background

The knowledge of subcellular location of proteins is fundamental for understanding their functions in biological processes [1]. In general, proteins must appear at right organelles in cells to transport signals and materials, catalyze metabolic reactions or provide structural support for cells. Mislocalization may affect these functions and lead to diseases, including cancers [2]. Colon cancer, a cancer type with the third highest morbidity and mortality across the world, has been found related to many subcellular translocations of proteins. For example, protein BCAR1 residing in cytoplasm and plasma membrane would transfer to nuclear in cancerous colon cells [3]. Other such proteins associated with colon cancer include EBP50 [4], TET2 [5], and beta-catenin [6]. Therefore, early detection of cancers can rely on not only the expression level of biomarker proteins [7], but also the change of protein subcellular locations between normal and malignant cells [8]. Nowadays, as the amount of protein data is huge and increases rapidly, automated subcellular location prediction is important for annotating new proteins and detecting protein translocations on a large scale.

In the past decades, lots of protein subcellular location prediction tools were developed, and some of them have been used in location biomarker analysis. Protein amino acid sequence, although ultimately determines the protein properties [9–11] and where the protein resides [12–15], is not a suitable data source for analyzing subcellular translocations because sequences generally do not change when the translocations occur [16]. In contrast, image-based methods that use immunohistochemistry (IHC) images can analyze the spatial distribution of proteins in normal and cancerous tissues and their location changes. Newberg and Murphy proposed a framework for analysis of protein spatial distribution, where subcellular location features (SLFs) were used to recognize protein subcellular patterns from IHC images, providing a starting point of applying IHC images to large-scale subcellular location prediction [17]; Xu et al. developed a multi-label subcellular location predictor named *i*Locator and applied it to location biomarker detection [16]; Kumar et al. proposed a pipeline to identify candidate cancer biomarkers by measuring whether the changes of protein expression level and subcellular location between normal and cancer tissues were significant [18]; Yang et al. recently built a protein subcellular localization predictor MIC_Locator, which transformed IHC images into frequency domain to capture local features and achieved high classification performance on multi-location proteins [19]. However, most of these statistical machine learning models used feature engineering that extracts predefined features to train classifiers. One disadvantage is that quality of models largely depends on the quality of features.

In recent years, the rise of deep learning provides another solution to study the protein spatial distributions. Some works based on convolutional neural networks (CNN) have been published, but most of these studies focus on the fluorescence images of cell lines [20–23], and cannot be used in detecting location biomarker proteins of cancerous tissues. Currently, only a few works tried to use deep learning methods on tissue images to analyze protein subcellular localization. Based on IHC images, Liu et al. proposed a classifier, SAE-RF, combining traditional statistical image features with a stacked auto-encoder [24]; and Long et al. designed a feature aggregator using deep neural networks with a multi-head self-attention mechanism [25]. These works have achieved good results on the protein localization task, but all of them used whole images as input of their deep models. This would lessen the capture of local subcellular patterns for deep neural networks as that IHC images show wide-field cell samples and have many non-informative sections.

In this work, we built an automated classifier of protein subcellular localization based on IHC images of colon tissue, and tested its ability of detecting protein translocation based on two constructed colon cancer biomarker datasets. The classifier used small image patches with high protein expression as model input, and combined both feature engineering and CNN models. Our results indicated that use of patches can improve the classification performance, and concatenating deep features and predefined features can be quite competitive in classifying subcellular localization of new samples. Proteins in the two biomarker datasets are collected from published literatures and the human protein atlas (HPA) database, respectively, and our classifier showed promising performance in identification of protein location changes.

## Results

**Fig. 1:**
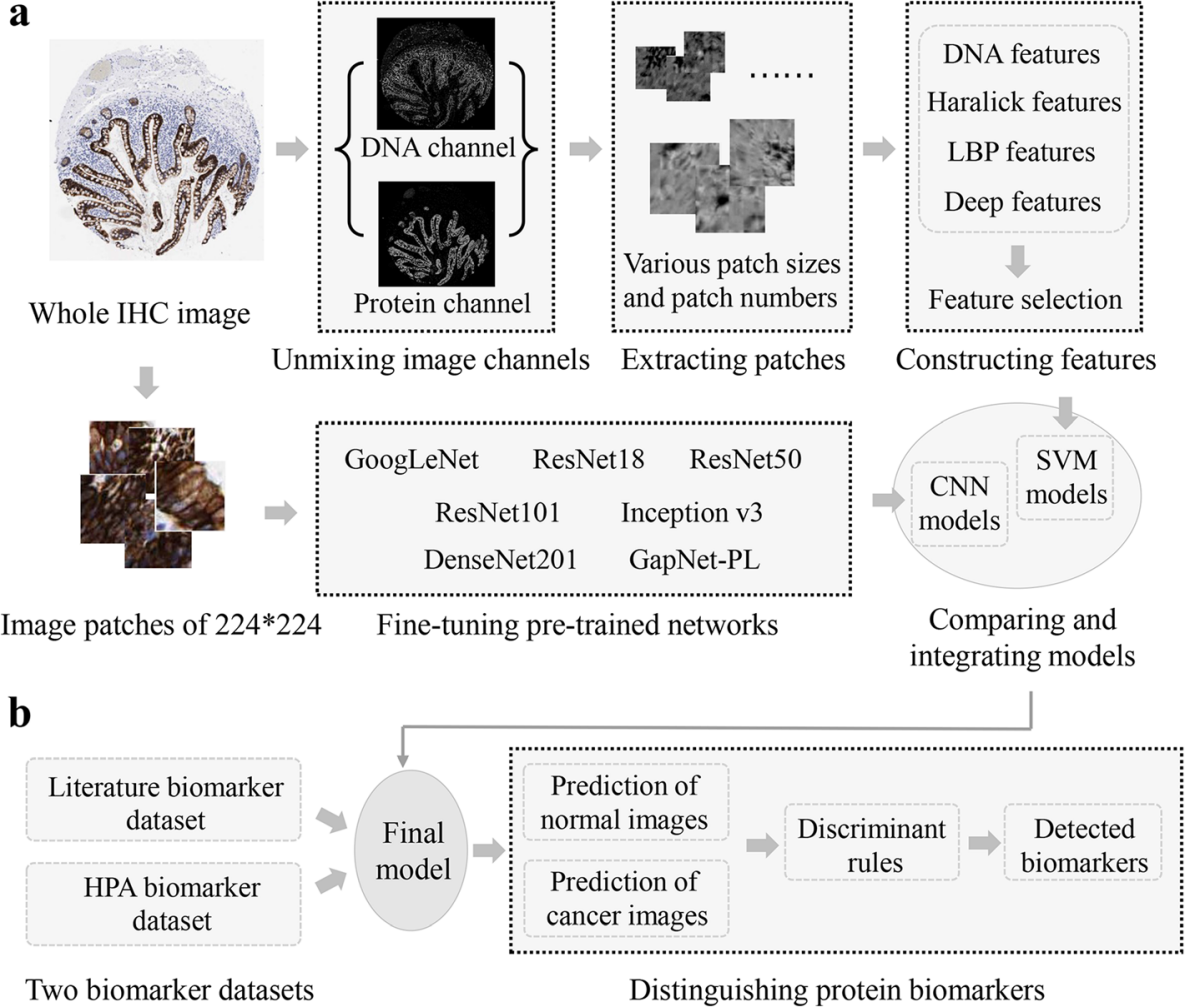
Framework of the experiments in this paper. **a** Training classifier models using IHC images. **b** Identifying location biomarker proteins using integrated model

Flow chart of our experiments is shown in Fig. 1. There are two stages, i.e., building classification models and distinguishing location biomarkers. In the first stage, an image-based protein subcellular localization model was built through combining feature engineering and deep learning methods. The feature engineering models were built through four steps, i.e., unmixing IHC image into protein and DNA channels, selecting interest image patches, extracting and selecting features, and training support vector machine (SVM) models (METHODS). Meanwhile, the selected interest patches were fed into deep CNN networks to fine-tune models and extract feature maps (METHODS). Then, a combined model was built by concatenating the features derived from the two pipelines and training a final SVM model. As one protein has 3 ~ 6 IHC images, two partition approaches were used to divide training and testing sets during model construction, i.e., per image and per protein. The former partition approach puts images into the training or testing set while the latter puts proteins into training or testing set. The difference is that the per protein way can ensure no overlap of proteins between training and testing set. In the second stage, we applied the model on the two biomarker datasets to test whether it can identify protein subcellular location changes, respectively.

### Classification results of using whole images

As a baseline, we firstly trained classification models based on the whole IHC images, where the patch extraction step was skipped. Each IHC image were unmixed using two methods, i.e., linear spectral unmixing (LIN) and blind spectral unmixing by non-negative matrix factorization (NMF), and then extracted global SLFs and local binary pattern (LBP) features. As SLFs extraction processed images by discrete wavelet transform using 10 Daubechies filters, we used db1 to db10 to represent different sets of features.

**Fig. 2:**
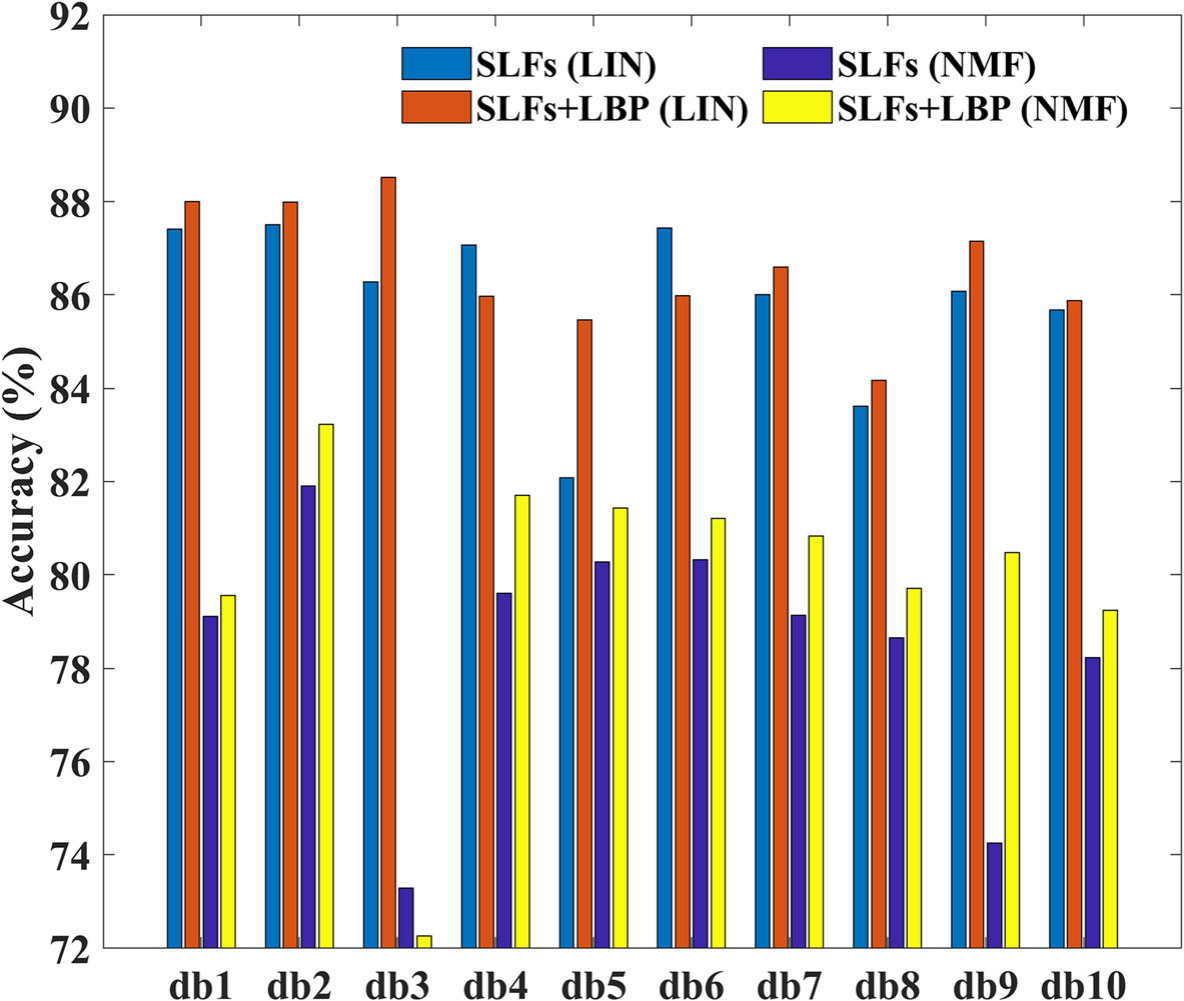
Classification results of using whole images with different image separation methods and features

Figure 2 shows the 10-fold cross validation results of using the two unmixing methods and whether using LBP features. These results are from experiments using the per image partition approach. It can be seen that the performance of the LIN approach outperformed NMF by 1.80 to 12.99% of accuracy when using only SLFs features. After adding the LBP features, the performance of LIN was better than NMF by 4.03 to 16.26% of accuracy. Besides accuracy, we also used recall, precision, and F1-score as evaluate metrics, and the three metrics showed similar results with accuracy (Table S-1). Since LIN produces better performance, which is consistent with conclusions in a previous study [17], all of subsequent experiments would use the LIN separation method to separate the DNA and protein channels. In addition, it seems that the classification performance benefit from the LBP features of whole images very slightly. This might be because that LBP features are more suitable for encoding local patterns, and are sensitive to the uninformative regions in images.

### Classification results of using interest patches

**Fig. 3:**
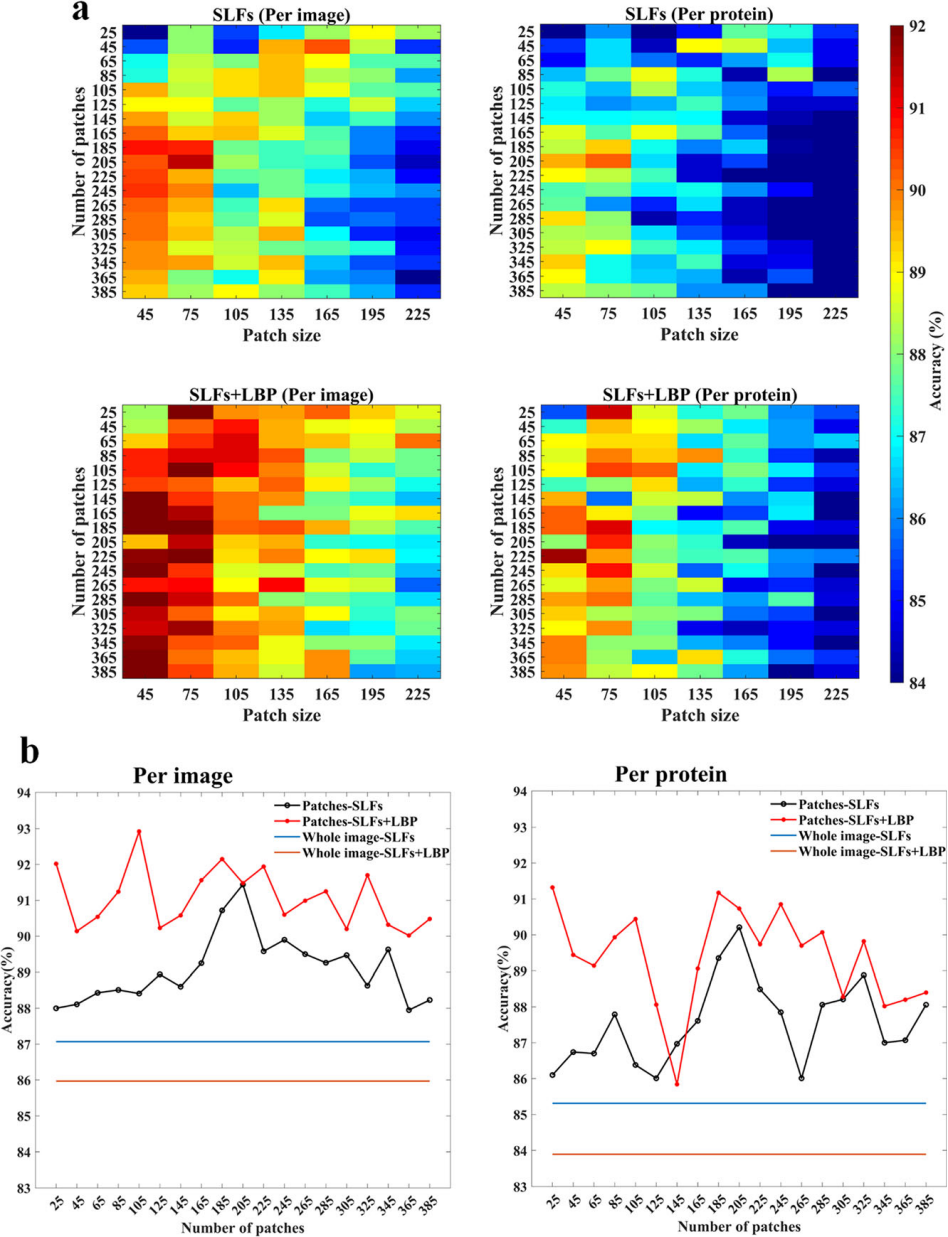
Classification results of using image patches. **a** Results of using different combinations of patch parameters. **b** Comparison of results between using whole images and using patches of 75*75 pixels. Features of db4 were used here

An original IHC image commonly consists of stained glands, unstained stroma, and unspecific background, but only the protein stained tissue section contains location pattern information. Therefore, we selected interest patches with high protein expression to represent subcellular patterns. It is assumed that two parameters, the number of patches in each image and the patch size, highly affect whether the patches contain enough and useful pattern information. Here, we set ranges for the two parameters and used grid search to determine the optimal ones. The number of patches was set from 25 to 385 in increments of 20, while the side length of the square patch was set from 45 to 225 pixels in increments of 30. Figure 3a shows the accuracy results of using different combinations of the two parameters.

**Fig. 4:**
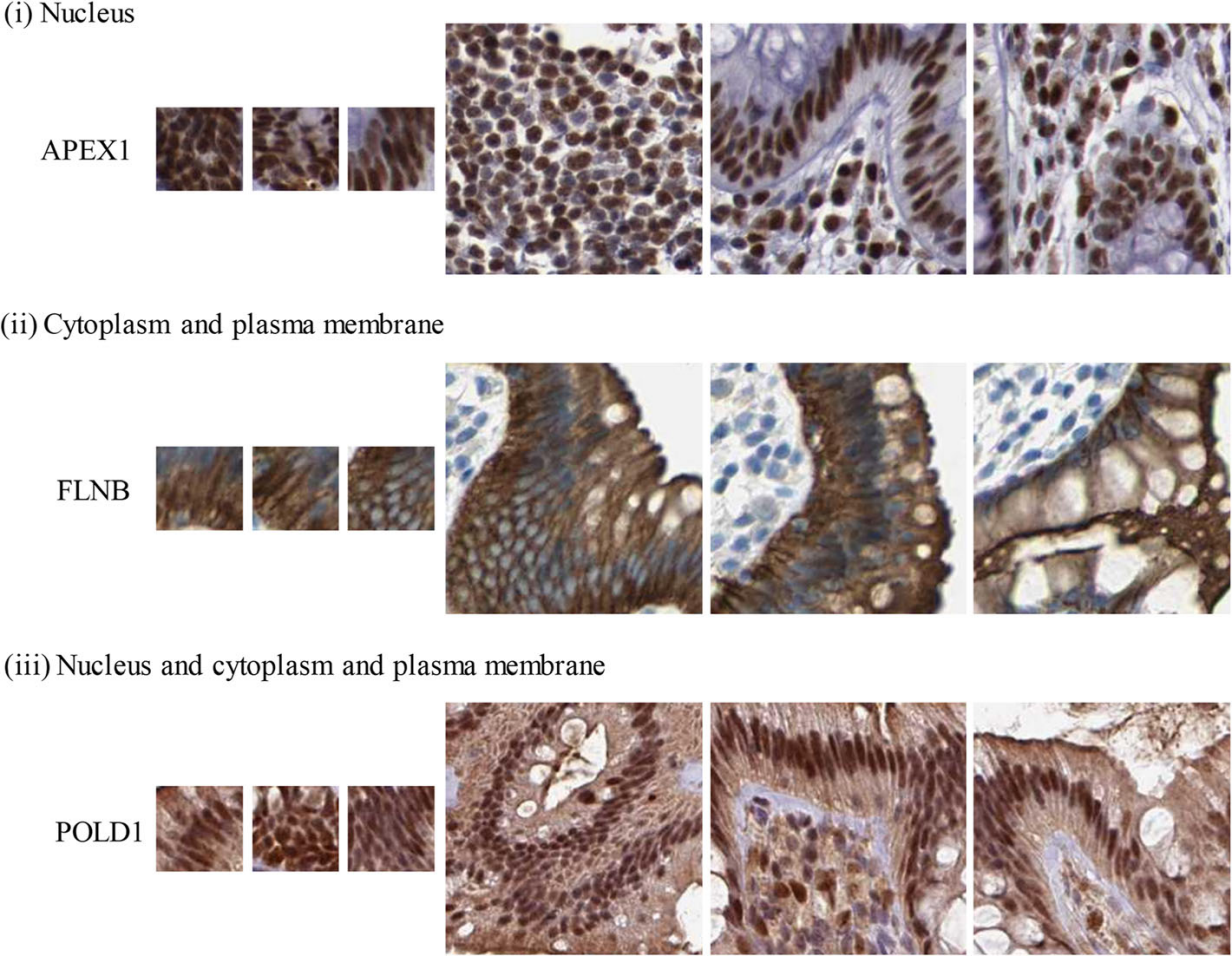
Examples of image patches of proteins in the three subcellular location classes. The patches in the left three columns have 75*75 pixels, while in the right three columns have 224*224 pixels

It can be seen from the heat maps that when the size of patches gets small and the number of regions increases, the classification accuracy gradually increases and then tends to be flat. Overall, the results below the main diagonal are better than the above. These results indicated that the patches should be sufficient to show protein patterns and be small to highlight the micropatterns in cells. Based on these heat maps, we selected optimal parameters for this classification task: the optimal size for the patches is 75 pixels and the optimal number of patches is 205. Some example patches with the optimal parameters are shown in Fig. 4.

Figure 3b compares the classification accuracies of using the whole images and patches, and results of other metrics are shown in Table S-2. It can be seen that using patches with appropriate parameters can achieve better results than directly using the features of whole images. Specifically, in the per image way, the results of selected patches outperform the whole image results by 0.88–4.37% when using the SLFs features, and by 4.05–6.95% when using the SLFs+LBP features. In the per protein way, the patch results outperform the whole image results by 0.7–4.9% if using the SLFs features, and by 1.94–7.42% if using the SLFs+LBP features. This also implies that LBP features play an important role in describing the subcellular location patterns in small image patches better than in images of wide vision, emphasizing the ability of LBP features to capture subtle local patterns.

In addition, we can see prediction accuracies in per protein way are lower than the per image way. This is because per protein way is a rigorous method for dataset partition, and it can objectively illustrate the generalization ability of trained models for new protein samples.

### Results of deep convolutional neural network models

As deep learning methods perform well in image classification, we tried to use convolutional neural networks to predict the subcellular location of proteins from IHC images. Seven pre-trained networks were used in the study, i.e., GoogLeNet [26], ResNet18 [27], ResNet50 [27], ResNet101 [27], Inception v3 [28], DenseNet201 [29] and GapNet-PL [30] (METHODS). To augment image data and grasp micropatterns of proteins, we extracted 26,705 patches with high protein expression from the images as the network inputs.

#### Classification results of using features from pre-trained networks

**Fig. 5:**
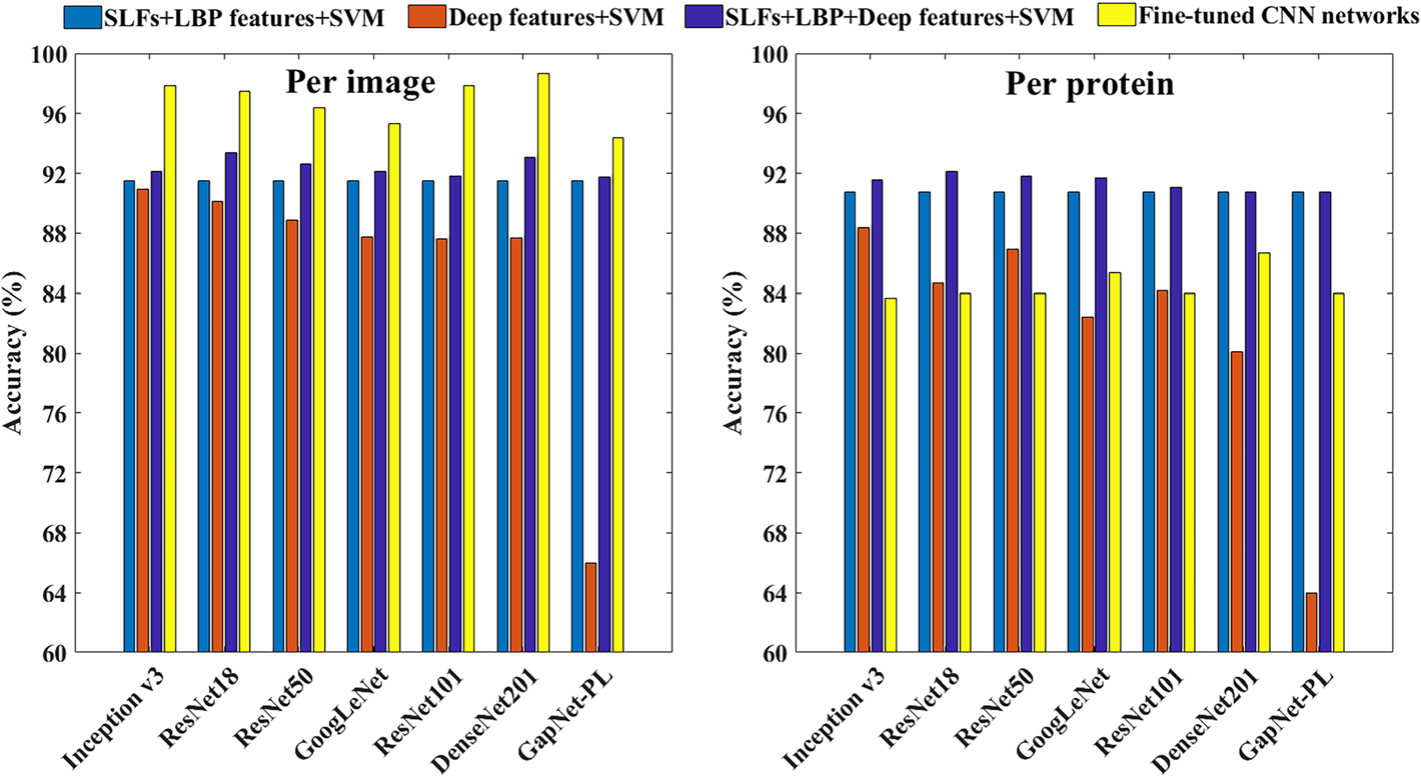
Classification results of SVM models and CNN models

We investigated the features extracted from the seven pre-trained networks. For each image, 35 patches of 224*224 pixels (Fig. 4) were extracted and fed into the networks to get feature maps. The features of patches in one image were averaged to obtain the image features, and fed into SVM models to perform a 10-fold cross-validation. The classification results using these features are shown as red bars in Fig. 5, where the Inception v3 network outperforms others. GapNet-PL shows bad performance probably because that it was a relatively shallow network designed for high-throughput fluorescence microscopy images, and the network structure cannot well identify the features in IHC images.

Then we attempted to combine the deep network features with SLFs+LBP features to see if the performance can be enhanced. It is shown as purple bars in Fig. 5 that concatenating the two types of features can achieve improved accuracies, especially for the GapNet-PL model. This is due to the robustness of SLFs+LBP features, which maintains the performance at a high level. Results of other evaluating metrics can be seen in Table S-3.

#### Classification results of fine-tuned deep neural networks

Besides, we also trained networks by fine tuning the seven pre-trained network models using the 26,705 image patches. The classification results are shown as yellow bars in Fig. 5. It can be seen that all the accuracies of networks in the per image way are above 94%, which is much better than the methods of feature engineering. However, the accuracies in the per protein way are quite low compared with the feature engineering models. This indicates that there might be overfitting in the per image models. As one protein may have different images in training and testing set when using the per image partition method, the results of per protein models are more objective in the method evaluation. The performances of all the pre-trained network models are very close, but there is a big gap in training time (Table S-4). For example, DenseNet201 model is superior to other networks in accuracy, but the training phase costs about 10 times longer than other models.

### Results of combined model

It can be concluded from Fig. 5 that concatenating SLFs, LBP, and the deep CNN features can achieve the best performance when using the per protein partition method. To build a final classifier with high classification and generalization performance, we concatenated SLFs, LBP features, and the feature maps of the seven networks together (each patch got a 7104-dimensional feature vector), selected 97 informative features by stepwise discriminant analysis, and then trained a final SVM model. Here, the feature maps were directly derived from the penultimate layers of the seven CNN models.

The combined model has better performance than all of the above single models. We compared its performance with four published models of IHC image-based protein subcellular localization, i.e., *i*Locator [16], SC-PSorter [31], MIC_ Locator [19], and SAE-RF [24] (Table 1). Among these models, *i*Locator studied the effects of local features and multi-label learning on classification of multi-locational proteins, while SC-PSorter introduced structural relationships among subcellular locations into models to enhance the performance. MIC_Locator used frequency features with different frequency scales to describe and classify protein subcellular patterns, while SAE-RF used conventional image features as input of 3-layer neural networks to distinguish subcellular patterns. All of them extract features from the whole IHC images. It can be seen that our method outperforms the other methods on all metrics, demonstrating that using image patches and integrating conventional features and deep features are effective in recognizing protein subcellular patterns.

**Table 1:** Comparison of our method with four existing protein location predictors

Method	Accuracy	Recall	Precision	F1-score
*i*Locator	76.16%	76.73%	81.57%	0.7908
SC-PSorter	78.81%	76.66%	86.22%	0.8040
MIC_Locator	79.69%	80.17%	86.71%	0.8291
SAE-RF	83.29%	85.57%	87.05%	0.8629
Our method	92.69%	93.55%	94.55%	0.9400

### Distinguishing protein biomarkers

The final classifier model was then applied to predicting the literature biomarker and HPA biomarker datasets to test its ability of distinguishing protein biomarkers (METHODS). Proteins in the two datasets have images of both normal and cancerous colons, and are likely to have different subcellular patterns between the two situations. Our model was expected to detect the differences. An independent sample *t*-test was performed based on the predicted score vectors, and the *P* values were used to assess the significance of location changes.

In the two biomarker datasets, one protein has 3 ~ 6 images of normal colon and 10 ~ 29 images of cancerous colon. For each protein, we determined its subcellular location of normal and cancer status by voting based on the outputs for images from the final models. Then, to generate more representative vectors for statistics, the output vectors of images of normal and cancer tissues from all the seven single classifiers trained on predefined and deep features are used to conduct independent sample *t*-test. In the literature biomarker dataset, 18 of the 22 proteins were detected as having significant location changes (Table S-5). Compared with the subcellular locations reported in literatures, the accuracy of the predicted subcellular locations in normal and cancer conditions are 68.18 and 40.19%, respectively. In the HPA biomarker dataset, 65.66% of the 795 proteins show significant location changes with *P* values less than 0.05 (Table S-5). The classification accuracies of subcellular locations of normal and cancer conditions are 84.36 and 84.66%, respectively. The results indicate that our model to some extent is able to distinguish the location changes of cancer biomarkers.

## Discussion

We have shown that the developed model benefits from the use of patches and combination of feature engineering and deep learning methods. The ability of the model in terms of detecting biomarker translocations was confirmed.

In this work, the IHC images were labeled as one of three subcellular location patterns (Fig. 4) according to annotations in the HPA database. We only considered the broad subcellular categories because IHC images showing tissue section are typically observed at cytoplasmic and nuclear levels, and fine-grained patterns in cells (for example, mitochondria and centrosome) can be hierarchically reflected in cytoplasm, nucleus or membrane. Another reason is that most of cancer biomarkers reported in literatures undergo translocations only among cytoplasm, nucleus, and plasma membrane.

We noted that our model was able to find subcellular location change between normal and cancer states. However, given the overall changes expected in visual appearance of cells in the comparison of cancer to normal tissue, there is a concern that most pairs of images would appear statistically different. Here we analyzed pairs of normal tissue images as a control. For each protein, we randomly split its normal tissue images into two sets, then used their model outputs to conduct independent sample *t*-test. It turned out that over 85% of the *P* values between normal images of the same proteins were larger than 0.05. It implies that our model might misclassify non-translocation cases as translocations when there is some certain variation in tissue structure or protein expression level. This could be improved in future work by better subcellular location classifiers and translocation discriminant rules.

**Fig. 6:**
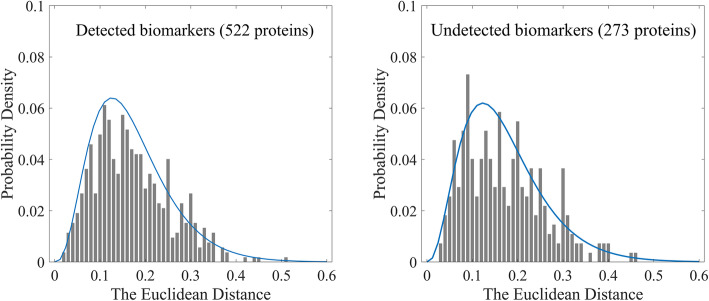
Comparison of intensity distance distribution between detected and undetected biomarkers in the HPA biomarker dataset

We also investigated whether the detected location changes between normal and cancer tissues came from the variation of image pixel distributions. For each protein in the HPA biomarker dataset, we represented its images by intensity distributions of their protein channels, and calculated Euclidean distances between all pairs of normal and cancer images. Then, the Euclidean distance distributions of those biomarkers detected by our method and of the biomarkers undetected were fitted by gamma distributions, respectively (Fig. 6). It can be seen that the two distributions are very similar, which indicates that the detection did not affected by image pixel distributions.

Some possible reasons that might underestimate the distinguishing ability of our model are listed as follows. First, some of the translocations of biomarkers in the literature biomarker dataset may only go for some subtypes of colon cancer. This is also the reason of the accuracy gap between normal and cancer conditions. Second, not all of the HPA biomarkers are sensitive for colon cancer. Even for those biomarkers of colon cancer, some of them would only change expression level in cancerous cells, which are not subcellular location biomarkers. This suggests us to consider both the protein expression level and subcellular location for biomarker detection in future works. Third, although the HPA database is a valuable source of protein spatial distribution, its manual subcellular locations may have errors and omissions because of biological variety and or human factors. This also would cause underestimate of our method.

## Conclusions

In this work, we established a bioimage-based classifier for protein subcellular localization, and used the classifier to reveal protein biomarkers. The classification results demonstrated that the image patches with proper parameters can achieve better performance than using the whole IHC images, and combining the traditional machine learning features with the neural network features is beneficial to the model performance. Besides, the application of the classifier to biomarker datasets indicates that our method can achieve satisfactory performance in location biomarker detection.

There is still room to improve for our method in future works. Firstly, the number of extracted patches from each image could be adaptive to the protein expression situation. The area of stained region is variable among images depending on the antibody binding and staining effect, so using a single optimal number fitting for all the images is difficult. We will attempt to use an adaptive patch number in future works, which is expected to lead to better performance.

Secondly, detection of cancer biomarker proteins should consider not only subcellular translocation, but also the change of expression level. Lots of proteins marked as cancer biomarkers in the HPA have unchanged subcellular location annotation in normal and cancerous tissues. These proteins may stay at normal locations and have abnormal expression level in cancerous cells. Therefore, in future studies we would use both protein expression levels and subcellular locations of proteins to analyze biomarkers, where the changes of protein staining and quantification in images can be additional information sources of cancer biomarkers.

## Methods

### Datasets

In this study, our image datasets were selected from the HPA (https://proteinatlas.org/) database, which is a public online database storing millions of IHC images of approximately 17,000 human proteins across various healthy and cancerous tissues [32]. Each IHC image in the database is a colored RGB image and has approximately 3000*3000 pixels. To ensure quality of data, we selected IHC images of proteins in colon tissue that fulfill three criteria: (a) the staining annotation was high or medium, (b) the intensity was annotated as strong or moderate, and (c) the quantity filed was annotated as greater than 25% [18]. According to annotations in the HPA, we put these images into three subcellular location classes, i.e., (i) nucleus, (ii) cytoplasm and plasma membrane, and (iii) nucleus and cytoplasm and plasma membrane (Fig. 4).

Three datasets were collected, i.e., modeling dataset, literature biomarker dataset, and HPA biomarker dataset, where the first was to build classifier models and the second and third were to validate the performance of the models on screening location biomarkers. The proteins in the literature biomarker dataset were collected for that they have been reported to transfer from one subcellular pattern to another in cancerous colon tissue (Table S-6), while the HPA biomarker dataset was composed of proteins that are marked as cancer biomarkers in the HPA. It is noted that the two validation datasets have no overlap with the modeling dataset. Details of the datasets can be seen in Table 2.

**Table 2:** Summary of the three datasets used in this study

Dataset	Number of proteins	Number of images		Number of patches		Number of images in each class		
		Normal	Cancer	Normal	Cancer	i	ii	iii
Modeling dataset	154	763	0	26,705	0	134	501	128
Literature biomarker dataset	22	111	659	3885	23,065	217	357	196
HPA biomarker dataset	795	2365	8351	82,775	298,585	1783	7683	1250

### Unmixing image channels

Since the distribution of proteins is a key factor in the classification, deriving protein channels from IHC images is a crucial step. Each IHC image in the HPA shows an immunohistochemically stained slide, where regions of a specific protein are stained brown by a monospecific antibody labeled with diaminobenzidine, and DNA in cell nuclei is stained purple by hematoxylin. We applied two color separation methods, i.e., LIN and NMF, to separate the protein and DNA channel. LIN uses one empirical color-base matrix to separate all the IHC images, while NMF calculated a unique color-base matrix for each image [17]. Both of the two methods can generate unmixed protein and DNA channels.

### Selecting patches from images

To extract informative patches, we performed a low-pass filter on the separated protein channels to select square patches of interest [18]. These patches generally have high level of protein expression, and are assumed to be able to represent the subcellular patterns of the whole images.

### Feature engineering classifiers

#### Feature extraction and selection

We extracted DNA features, Haralick texture features, and LBP features to describe the subcellular location of proteins [33]. Sixteen dimensional DNA features related to the protein and nuclear overlap and distance were extracted. Haralick texture features were extracted from the gray level co-occurrence matrices of images. In this study, we extracted the Haralick features using 10 Daubechies filters with vanishing moment from 1 to 10, each of which had 576-dimensional features. DNA and Haralick features are a subset of global SLFs identified by Murphy group. In addition, we also extracted 256 dimensional LBP features, which can describe the spatial structure of local patterns and can detect microscopic textures in images. In total, there are 848 features for each patch, including 592 global SLFs and 256 LBP features.

Considering high-dimensional features may cause overfitting and lead to poor generalization of classifiers, we used a feature selection method, stepwise discriminant analysis, to reduce dimensionality, as it has been proven to be superior to other feature selection methods in subcellular image classification [34].

#### Classifier design

We used SVM from LIBSVM-3.23 toolbox (https://csie.ntu.edu.tw/~cjlin/libsvm) with radial basis function kernel to train classifier models [35], and the parameter *g* and *c* were determined by grid search. 10-fold cross validation was employed here to evaluate the model performance.

### Deep convolutional neural network models

#### Pre-trained networks

Seven pre-trained networks were used in the study. The first six networks are GoogLeNet [26], ResNet18 [27], ResNet50 [27], ResNet101 [27], Inception v3 [28], and DenseNet201 [29], all of which were trained by a massive amount of natural images in the ImageNet database [36]. These architectures have robust performance in image feature representation and have been widely used in many transfer learning works [37, 38]. The last one is GapNet-PL, which was a network architecture designed to process high-throughput fluorescence microscopy images and predict protein subcellular location patterns [30]. The network consists of 8 convolutional layers, 5 pooling layers, and 3 fully-convolutional layers. The outstanding characteristic of this structure is that feature maps of three different layers are reshaped to a size of one pixel by global average pooling, and then the concatenated feature vector is passed to a fully connected layer for prediction. The pooling operation can connect feature information from different levels and greatly reduce the number of parameters. In addition, the network replaces ReLU and batch normalization with SELU activation function, which significantly reduces the training time of the model and lower memory consumption. Compared with other models using ReLU and batch normalization, the F1-score was improved by 2–6% [30].

#### Transfer learning from the pre-trained networks

We operated two methods of transfer learning, i.e., extracting feature map from pre-trained networks and fine-tuning method. Firstly, the penultimate layers of the seven networks were extracted as patch features, where the GapNet-PL outputs 256 features, while the other six networks output 1000 features. Besides, fine-tuning was used on the pre-trained networks to adapt the classification models to our task. For each network, we replaced the last layer with our classification outputs, and fine-tuned the parameters of all the layers.

### Distinguishing protein biomarkers

In this work, we used two biomarker datasets, i.e., literature biomarker dataset and HPA biomarker dataset, to verify whether the machine learning models can detect protein translocations in colon cancer. Independent sample *t*-test was used to evaluate the significance of location changes. In particular, suppose one protein has *m* images of normal tissue and *n* images of cancer tissue. First, we used the averaged classification score vectors to determine the predictions of subcellular location in normal and cancer tissues, respectively. Then for each protein, an independent sample *t*-test was conducted under a null hypothesis that the mean vectors are the same between the *m* weight vectors of the normal tissue images and the *n* weight vectors of the cancer tissue images. The *t*-test would output a *P* value vector, where each value indicated the significance of change of subcellular location from normal to cancer status. Protein was considered to be identified as location biomarkers only if the *P* value is less than 0.05.

## Supplementary information

**Additional file 1: Table S1.** Results of using different image separation methods and features. **Table S2.** Comparison of using whole images and using patches. **Table S3.** Results of combining conventional and deep learning features. **Table S4.** Training time of fine tuning pre-trained deep networks. **Table S6.** Subcellular location changes of proteins in the literature biomarker dataset.

**Additional file 2: Table S5.** Predicted subcellular locations and *P* values of proteins in the literature biomarker dataset and the human protein atlas biomarker dataset.

## Data Availability

The datasets and code used in this study are available at https://github.com/Xue-zhen-zhen/Protein-subcellular-location

## Abbreviations

IHC: Immunohistochemistry
SLFs: Subcellular location features
CNN: Convolutional neural networks
SAE-RF: Stacked auto encoder - Random forest
HPA: Human protein atlas
SVM: Support vector machine
LIN: Linear spectral unmixing
NMF: Blind spectral unmixing byNon-negative matrix factorization
LBP: Local Binary Patterns
ReLU: Rectified linear unit
SELU: Scaled exponential linear units
