# Supplementary material for: Automated classification of protein subcellular localization in immunohistochemistry images to reveal biomarkers in colon cancer
Source: BMC Bioinformatics. 2020 Sep 9;21:398. doi: 10.1186/s12859-020-03731-y (PMC7487883; doi:10.1186/s12859-020-03731-y)
Supplement: Supplementary file 1 — Additional file 1: Table S1. Results of using different image separation methods and features. Table S2. Comparison of using whole images and using patches. Table S3. Results of combining conventional and deep learning features. Table S4. Training time of fine tuning pre-trained deep networks. Table S6. Subcellular location changes of proteins in the literature biomarker dataset. [file 12859_2020_3731_MOESM1_ESM.docx]

**SUPPLEMENTARY DATA FOR**

Automated classification of protein subcellular localization in immunohistochemistry images to reveal biomarkers in colon cancer

*Zhen-Zhen Xue*^1,6^, *yanxia Wu^2,3,6^, Qing-Zu Gao^4^, Liang Zhao^2,5^ and Ying-Ying Xu*^1*^

^1^ School of Biomedical Engineering and Guangdong Provincial Key Laboratory of Medical Image Processing, Southern Medical University, Guangzhou 510515, China ^2^ Department of Pathology, Nanfang Hospital, Southern Medical University, Guangzhou 510515, China ^3^ Department of Clinical Pathology, Affiliated Hospital of Guangdong Medical University, Zhanjiang 524000, China ^4^ The First Affiliated Hospital of Xinxiang Medical University, Xinxiang, China ^5^ Department of Pathology, School of Basic Medical Sciences, Southern Medical University, Guangzhou 510515, China ^6^ These authors contributed equally

*Correspondence: yyxu@smu.edu.cn

Table of contents

Table S-1. Results of using different image separation methods and features.

Table S-2. Comparison of using whole images and using patches.

Table S-3. Results of combining conventional and deep learning features.

Table S-4. Training time of fine tuning pre-trained deep networks.

Table S-6. Subcellular location changes of proteins in the literature biomarker dataset.

Reference

**Table S-1.** Results of using different image separation methods and features. Only experimental results of db4 features are shown.

| Evaluation metrics  Feature types | | | Accuracy | Recall | Precision | F1-score |
| --- | --- | --- | --- | --- | --- | --- |
| Per image | LIN | SLFs | 87.07% | 88.69% | 90.39% | 0.8941 |
|  |  | SLFs+LBP | 85.97% | 87.26% | 89.28% | 0.8817 |
|  | NMF | SLFs | 79.60% | 80.70% | 84.58% | 0.8332 |
|  |  | SLFs+LBP | 81.70% | 82.58% | 86.56% | 0.8424 |
| Per protein | LIN | SLFs | 85.31% | 86.53% | 89.00% | 0.8752 |
|  |  | SLFs+LBP | 83.90% | 85.26% | 87.40% | 0.8614 |
|  | NMF | SLFs | 76.56% | 78.04% | 81.45% | 0.7923 |
|  |  | SLFs+LBP | 79.77% | 79.88% | 84.85% | 0.8182 |

**Table S-2.** Comparison of using whole images and using patches. Only experimental results of db4 features on images and patches (patch size is 75*75 pixels and each image has 205 patches) are shown.

| Evaluation metrics  Feature types | | | Accuracy | Recall | Precision | F1-score |
| --- | --- | --- | --- | --- | --- | --- |
| Per image | Whole image | SLFs | 87.07% | 88.69% | 90.39% | 0.8941 |
|  |  | SLFs+LBP | 85.97% | 87.26% | 89.28% | 0.8817 |
|  | Patches | SLFs | 91.44% | 92.91% | 93.31% | 0.9309 |
|  |  | SLFs+LBP | 91.48% | 92.91% | 93.89% | 0.9337 |
| Per protein | Whole image | SLFs | 85.31% | 86.53% | 89.00% | 0.8752 |
|  |  | SLFs+LBP | 83.90% | 85.26% | 87.40% | 0.8614 |
|  | Patches | SLFs | 90.21% | 91.08% | 92.49% | 0.9166 |
|  |  | SLFs+LBP | 90.73% | 92.08% | 92.35% | 0.9215 |

**Table S-3.** Results of combining conventional and deep learning features. Conventional features are SLFs+LBP features.

|  | Pre-trained networks | Accuracy | Recall | Precision | F1-score |
| --- | --- | --- | --- | --- | --- |
| Per image | Inception-v3 | 92.16% | 93.31% | 94.72% | 0.9396 |
|  | ReseNet18 | 93.39% | 94.38% | 95.23% | 0.9477 |
|  | ReseNet50 | 92.64% | 94.05% | 94.65% | 0.9432 |
|  | GoogLeNet | 92.12% | 93.31% | 94.22% | 0.9374 |
|  | ReseNet101 | 91.82% | 92.89% | 94.08% | 0.9346 |
|  | DenseNet201 | 93.06% | 94.19% | 94.72% | 0.9442 |
|  | GapNet-PL | 91.77% | 93.19% | 94.10% | 0.9362 |
| Per protein | Inception-v3 | 91.55% | 93.05% | 93.87% | 0.9343 |
|  | ReseNet18 | 92.12% | 92.69% | 93.61% | 0.9306 |
|  | ReseNet50 | 91.81% | 92.02% | 93.11% | 0.9252 |
|  | GoogLeNet | 91.68% | 92.09% | 92.94% | 0.9244 |
|  | ReseNet101 | 91.05% | 92.18% | 92.60% | 0.9232 |
|  | DenseNet201 | 90.74% | 91.91% | 92.87% | 0.9232 |
|  | GapNet-PL | 90.76% | 92.04% | 92.77% | 0.9256 |

**Table S-4.** Training time of fine tuning pre-trained deep networks. All the times are calculated on a Quadro M4000 GPU.

| Pre-trained network models | Training time (h) |
| --- | --- |
| DenseNet201 | 106.10 |
| GapNet-PL | 29.80 |
| Inception v3 | 16.50 |
| ResNet101 | 13.30 |
| ResNet50 | 7.30 |
| GoogLeNet | 4.20 |
| RenNet18 | 3.80 |

**Table S-6.** Subcellular location changes of proteins in the literature biomarker dataset.

| Proteins | Subcellular location | | References |
| --- | --- | --- | --- |
|  | Normal tissue | Cancer tissue |  |
| p53* | Nucleus | Nucleus; Cytoplasm | [1] |
| EBP50* | Cytoplasm | Nucleus | [2] |
| CDKN1B* | Nucleus | Cytoplasm | [3] |
| NDRG1 | Cytoplasm | Plasma membrane; Nucleus | [4] |
| beta-catenin* | Plasma membrane | Cytoplasm; Nucleus | [5] |
| p68* | Nucleus | Cytoplasm | [6] |
| p130Cas* | Cytoplasm; Plasma membrane | Nucleus | [7] |
| CCNEL | Cytoplasm; Plasma membrane | Nucleus | [8] |
| CYSLT1 | Plasma membrane | Nucleus; Plasma membrane | [9] |
| STAT3* | Cytoplasm | Nucleus | [10] |
| PRKCA* | Cytoplasm | Plasma membrane | [11] |
| TET2* | Nucleus | Cytoplasm ; Nucleus | [12] |
| CACYBP* | Cytoplasm | Nucleus | [13] |
| PRKCB* | Cytoplasm | Plasma membrane | [14] |
| SMAD3* | Cytoplasm | Nucleus | [15] |
| BRD4* | Cytoplasm | Nucleus | [16] |
| BCL2 | Cytoplasm; Nucleus; Plasma membrane | Cytoplasm | [17] |
| ELAVL1* | Nucleus | Cytoplasm | [18] |
| HNRNPK* | Nucleus | Cytoplasm ; Nucleus | [19] |
| AHR* | Cytoplasm | Nucleus | [20] |
| AKT1* | Nucleus | Cytoplasm; Nucleus | [21] |
| ARRB1* | Cytoplasm | Plasma membrane | [22] |

‘*’ indicates the proteins that can be detected as biomarkers by our approach.

Reference

1. Bosari S, Viale G, Roncalli M, Graziani D, Borsani G, Lee A, Coggi G. p53 gene mutations, p53 protein accumulation and compartmentalization in colorectal adenocarcinoma. The American journal of pathology. 1995; 147(3):790-798.

2. Lin Y-Y, Hsu Y-H, Huang H-Y, Shann Y-J, Huang C-YF, Wei S-C, Chen C-L, Jou T-S. Aberrant nuclear localization of EBP50 promotes colorectal carcinogenesis in xenotransplanted mice by modulating TCF-1 and β-catenin interactions. The Journal of clinical investigation. 2012; 122(5):1881-1894.

3. Ogino S, Shima K, Nosho K, Irahara N, Baba Y, Wolpin BM, Giovannucci EL, Meyerhardt JA, Fuchs CS. A cohort study of p27 localization in colon cancer, body mass index, and patient survival. Cancer Epidemiology and Prevention Biomarkers. 2009; 18(6):1849-1858.

4. Song Y, Lv L, Du J, Yue L, Cao L. Correlation of N-myc downstream-regulated gene 1 subcellular localization and lymph node metastases of colorectal neoplasms. Biochem Biophys Res Commun. 2013; 439(2):241-246.

5. Yeo M, Kim DK, Park HJ, Oh TY, Kim JH, Cho SW, Paik YK, Hahm KB. Loss of transgelin in repeated bouts of ulcerative colitis-induced colon carcinogenesis. Proteomics. 2006; 6(4):1158-1165.

6. Shin S, Rossow KL, Grande JP, Janknecht R. Involvement of RNA helicases p68 and p72 in colon cancer. Cancer Res. 2007; 67(16):7572-7578.

7. Casanova I, Parreno M, Farre L, Guerrero S, Cespedes MV, Pavon MA, Sancho FJ, Marcuello E, Trias M, Mangues R. Celecoxib induces anoikis in human colon carcinoma cells associated with the deregulation of focal adhesions and nuclear translocation of p130Cas. Int J Cancer. 2006; 118(10):2381-2389.

8. LaQuaglia MJ, Grijalva JL, Mueller KA, Perez-Atayde AR, Kim HB, Sadri-Vakili G, Vakili K. YAP Subcellular Localization and Hippo Pathway Transcriptome Analysis in Pediatric Hepatocellular Carcinoma. Sci Rep. 2016; 6:30238-30238.

9. Nielsen CK, Campbell JI, Öhd JF, Mörgelin M, Riesbeck K, Landberg G, Sjölander A. A novel localization of the G-protein-coupled CysLT1 receptor in the nucleus of colorectal adenocarcinoma cells. Cancer research. 2005; 65(3):732-742.

10. Lassmann S, Schuster I, Walch A, Gobel H, Jutting U, Makowiec F, Hopt U, Werner M. STAT3 mRNA and protein expression in colorectal cancer: effects on STAT3-inducible targets linked to cell survival and proliferation. J Clin Pathol. 2007; 60(2):173-179.

11. Ko C-H, Shen S-C, Lee TJ, Chen Y-C. Myricetin inhibits matrix metalloproteinase 2 protein expression and enzyme activity in colorectal carcinoma cells. Molecular cancer therapeutics. 2005; 4(2):281-290.

12. Huang Y, Wang G, Liang Z, Yang Y, Cui L, Liu CY. Loss of nuclear localization of TET2 in colorectal cancer. Clin Epigenetics. 2016; 8(1):9-9.

13. Feng S, Zhou Q, Yang B, Li Q, Liu A, Zhao Y, Qiu C, Ge J, Zhai H. The effect of S100A6 on nuclear translocation of CacyBP/SIP in colon cancer cells. PLoS One. 2018; 13(3):e0192208.

14. Shah SA, Looby E, Volkov Y, Long A, Kelleher D. Ursodeoxycholic acid inhibits translocation of protein kinase C in human colonic cancer cell lines. Eur J Cancer. 2005; 41(14):2160-2169.

15. Matsushita M, Matsuzaki K, Date M, Watanabe T, Shibano K, Nakagawa T, Yanagitani S, Amoh Y, Takemoto H, Ogata N. Down-regulation of TGF-β receptors in human colorectal cancer: implications for cancer development. British journal of cancer. 1999; 80(1-2):194-205.

16. You JS, Jones PA. Cancer genetics and epigenetics: two sides of the same coin? Cancer Cell. 2012; 22(1):9-20.

17. Sinicrope FA, Cleary KR, Stephens LC, Lee JJ, Levin B. bcl-2 and p53 oncoprotein expression during colorectal tumorigenesis. Cancer research. 1995; 55(2):237-241.

18. Lim SJ, Lee SH, Joo SH, Song JY, Choi SI. Cytoplasmic expression of HuR is related to cyclooxygenase-2 expression in colon cancer. Cancer Res Treat. 2009; 41(2):87-92.

19. Carpenter B, McKay M, Dundas SR, Lawrie LC, Telfer C, Murray GI. Heterogeneous nuclear ribonucleoprotein K is over expressed, aberrantly localised and is associated with poor prognosis in colorectal cancer. Br J Cancer. 2006; 95(7):921-927.

20. Johnson JJ, Mukhtar H. Curcumin for chemoprevention of colon cancer. Cancer Lett. 2007; 255(2):170-181.

21. Rychahou PG, Kang J, Gulhati P, Doan HQ, Chen LA, Xiao SY, Chung DH, Evers BM. Akt2 overexpression plays a critical role in the establishment of colorectal cancer metastasis. Proc Natl Acad Sci U S A. 2008; 105(51):20315-20320.

22. Buchanan FG, Gorden DL, Matta P, Shi Q, Matrisian LM, DuBois RN. Role of beta-arrestin 1 in the metastatic progression of colorectal cancer. Proc Natl Acad Sci U S A. 2006; 103(5):1492-1497.
